# Use of Whole-Exome Sequencing and Pedigree Analysis to Identify X-linked Hypophosphatemia in Saudi Arabian Families

**DOI:** 10.1210/jendso/bvae203

**Published:** 2024-11-18

**Authors:** Mohamed H Al-Hamed, Sarah Bakhamis, Sara I Abdelfattah, Afaf Alsagheir

**Affiliations:** Department of Clinical Genomics, Center for Genomic Medicine, King Faisal Specialist Hospital and Research Center, Riyadh, 11564, Saudi Arabia; College of Medicine, Alfaisal University, Riyadh, 11211, Saudi Arabia; College of Medicine, Alfaisal University, Riyadh, 11211, Saudi Arabia; Department of Pediatrics, King Faisal Specialist Hospital and Research Center, Riyadh, 11533, Saudi Arabia; College of Medicine, Alfaisal University, Riyadh, 11211, Saudi Arabia; Department of Pediatrics, King Faisal Specialist Hospital and Research Center, Riyadh, 11533, Saudi Arabia

**Keywords:** X-linked hypophosphatemia, whole-exome sequencing, consanguinity, PHEX, genetic testing, Saudi Arabia

## Abstract

**Context:**

X-linked hypophosphatemia (XLH) is the most common form of inherited hypophosphatemic rickets (HR), caused by pathogenic variants in the *PHEX* gene. Genetic diagnosis of XLH facilitates early treatment optimization, especially for patients suitable for burosumab, a recombinant anti-fibroblast growth factor-23 monoclonal antibody.

**Objective:**

This study aimed to use whole-exome sequencing (WES) and pedigree analysis to identify patients with XLH.

**Methods:**

Medical records at a single center in Saudi Arabia were screened between 2014 and 2024 to identify patients with suggested HR. Of the 800 patients identified, 27 had had suspected XLH. The genetic study comprised 100 patients drawn from these 27 families.

**Results:**

Clinical manifestations were widespread and variable within families. Severe disease was reported in 55% of children and 25% of adults. At presentation, all children were receiving either conventional therapy (60%) or burosumab (40%); however, 53% of adults were not treated. WES provided a genetic diagnosis in 23 families: alterations in the *PHEX* gene (20 families), with homozygous *ENPP1* and *DMP1* variants detected in 2 and 1 families, respectively. Pathogenic/likely pathogenic variants were detected in 23 families (diagnostic yield 85%). Ten novel likely pathogenic variants were detected. Pedigree analysis provided information to support disease-specific patient management.

**Conclusion:**

WES detected a diagnostic molecular abnormality in 85% of families with HR phenotypes; *PHEX* variants were the most common. Combined use of WES and pedigree analysis highlighted the underdiagnosis of adult XLH in this population, with most family members being diagnosed after the pedigree analysis.

Hypophosphatemic rickets (HR) is a disorder of bone mineralization resulting from inherited or acquired defects in the renal handling of phosphorus. Nutritional (acquired) rickets remains prevalent in the Arab countries [1] and was reported as the most common cause of rickets and osteomalacia in children and adolescents attending an endocrine clinic in Riyadh, Saudi Arabia [2]. Because of the high rate of consanguinity in the region, nutritional rickets may coexist with inherited forms of rickets [1]. The most common cause of inherited HR and osteomalacia is X-linked hypophosphatemia (XLH) [3], a rare genetic disorder affecting 1 in 20 000 individuals worldwide [4]. In Saudi Arabia, a high index of suspicion is required to accurately diagnose the rare forms of rickets [1, 5] and provide effective treatment early in the disease course. XLH is a phenotypically heterogeneous disease with numerous multisystem clinical manifestations, the severity of which are highly variable even among affected individuals within the same family [3, 6-9].

The phosphate-regulating endopeptidase homolog (*PHEX*) gene, located on the X chromosome [10, 11], is responsible for regulating the expression of fibroblast growth factor 23 (FGF23). Elevated levels of FGF23 result in impaired renal reabsorption of phosphate and downregulation of 1alpha-hydroxylase activity, ultimately leading to the symptoms associated with XLH and related conditions including Fanconi syndrome and hereditary hypophosphatemic rickets with hypercalciuria [6]. Approximately 63% to 89%% of inherited HR cases are due to pathogenic *PHEX* variants and result in XLH; the remaining cases are due to mutations in other genes involved in the synthesis, signaling, and regulation of FGF23, including *FGF23*, *ALPL*, *CYP27B1*, *DMP1*, and *ENPP1* [3]. Notably, a substantial minority of *PHEX* mutations may be missed by whole-exome sequencing (WES) because they are deep intronic mutations [3].

With the recent advent of burosumab, the first disease-modifying therapy approved to treat XLH by inhibiting FGF23 to restore phosphate homeostasis, efforts to improve the early diagnosis of patients with XLH are needed [12]. Early diagnosis is crucial to enable treatment initiation at an early age and thereby optimize long-term outcomes [12].

The aim of this study was to use genetic pedigree analysis and WES to identify the molecular genetic causes of patients with HR, identify and characterize those patients with XLH, and enable the proactive identification of previously undiagnosed symptomatic and asymptomatic patients with XLH within affected families.

## Materials and Methods

### Study Cohort

Healthcare Information Technology Affairs indexed records at the King Faisal Specialist Hospital and Research Centre (KFSHRC) were screened for a 10-year period between December 2014 and January 2024 to identify patients with suggested HR (ie, patients with low phosphate levels and manifestations suggestive of rickets). A total of 800 patients with suggested HR were identified. Individual records were reviewed to identify those with suspected XLH based on the following criteria: alkaline phosphatase [ALP] above normal for age, low serum phosphate, low or inappropriate normal serum 1,25(OH)_2_ vitamin D and high urine phosphate level, and/or a family history of XLH. A total of 20 patients were identified with suspected XLH. In addition to these 20 patients, a further 7 were identified through pediatric endocrinology and orthopedic clinic visits at the same hospital.

**Figure 1.**
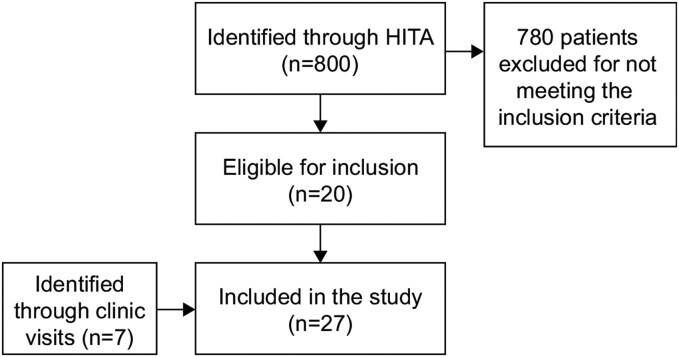
Flow diagram of patient recruitment. Abbreviation: HITA, Healthcare Information Technology Affairs.

The families of the 27 patients with suspected XLH were contacted to confirm their participation in and consent for the genetic study. A total of 100 patients, representing the 27 families, were included in the study (Fig. 1). Each family was contacted for a detailed family history, and the parents and each symptomatic family member were invited to the clinic for initial screening, including typical signs and symptoms of XLH (ie, short stature, bowing of the lower limbs, having a previous surgery, and suffering from chronic pains and aches), basic biochemical markers (ALP, phosphate, calcium, PTH), and an X-ray of the spine and lower limbs. Pedigree charts were drawn from which the total number of affected family members was calculated. One affected patient from each family consented to undergo DNA genetic testing through WES. All 100 patients underwent genetic testing, starting with the index test; Sanger sequencing validation was then performed to confirm the familial segregation of the detected variants in all family members. The study was conducted according to the Declaration of Helsinki and approved by the local institutional review board (KFSHRC RAC# 2211035). DNA was extracted using a Gentra Systems PUREGENE DNA Isolation kit (Qiagen, USA).

### Clinical Assessment

Clinical assessment was performed by trained clinicians at the KFSHRC, the largest hospital system and national specialist center of excellence for XLH and related conditions in the Kingdom of Saudi Arabia. Height and weight were measured by trained nurses on the same scales in the same clinic at the Center. Short stature was defined if height was less than 2 SDs for sex, age, and ethnicity using the national Saudi Arabian growth chart [13]. Limb bowing was assessed clinically by specialist physicians. Bowing was noted if the feet and ankles were together and the knees remained apart or if the knees were together and the feet and ankles were apart. Laboratory tests were performed in the chemical pathology laboratory at KFSHRC using standard methods. Reference values are given in the tables below and are based on the standard reference values used at KFSHRC. X-rays were evaluated by specialist radiologists who were blinded to disease status. Craniosynostosis was noted during physical examination by specialist pediatricians or adult physicians. Cases of craniosynostosis were referred to the neurosurgery department, which typically arranged 3-dimensional computed tomography for further assessment and management.

Disease severity classification criteria are described in Table 1.

**Table 1.** Baseline characteristics of study participants (n = 100)

Patient status at time of screening	Overall number/total (%)		
Alive	96 (96%)		
Deceased	4 (4%) None of the deaths were related to complications associated with HR		
**Clinical manifestation at presentation**	***Children*** number/total (%)	***Adults*** number/total (%)	***Overall*** number/total (%)
Short stature	38/43 (88%)	56/57 (98%)	94/100 (94%)
Bowing	38/43 (88%)	48/57 (84%)	86/100 (86%)
Craniosynostosis	14/43 (30%)	7/57 (12%)	21/100 (21%)
Asymptomatic	0	1/57 (2%)	1/100 (1%)
**Disease severity** *^a^*	***Children*** number/total (%)	***Adults*** number/total (%)	***Overall*** number/total (%)
Mild	0	2/57 (4%)	2 (2%)
Moderate	19/43 (44%)	39/57 (68%)	58 (58%)
Severe	24/43 (55%)	15/57 (26%)	39 (39%)
**Treatment at time of presentation**	***Children*** number/total (%)	***Adults*** number/total (%)	***Overall*** number/total (%)
Burosumab	18/43 (42%)	1/57 (2%)	32 (32%)
Conventional	25/43 (58%)	26/57 (46%)	51 (51%)
No treatment	0	30/57 (53%)	30 (30%)

Abbreviation: HR, hypophosphatemic rickets.

^*a*^Disease severity was defined as: Mild: only biochemical abnormalities suggestive of HR (ie, low phosphate and high alkaline phosphatase for age and gender) and otherwise asymptomatic with no skeletal deformity. Moderate: biochemical abnormalities suggestive of HR with skeletal deformity. Severe: biochemical abnormalities suggestive of HR with skeletal deformity and pain with limitation in mobility ± using a mobility assistance device.

### Ethical Approval and Consent

The study was approved by the Research Advisory Council at KFSHRC, Riyadh, Saudi Arabia (KFSHRC RAC# 2211035). The study was conducted in accordance with the local legislation and institutional requirements. Written informed consent for participation in this study was provided by the participants’ legal guardians/next of kin. Written informed consent was obtained from the individual(s), and minor(s)’ legal guardian/next of kin, for the publication of any potentially identifiable images or data included in this article.

### WES and Variant Interpretation

WES was performed on the patients’ genomic DNA as described previously [14]. In brief, DNA libraries were constructed using an Agilent Sureselect All Exons V6 (50 Mb) capture kit and sequenced on an Illumina NovaSeq 6000 platform with an average target depth of 80×. Exome data were mapped to the human reference genome (NCBI build 37.1, UCSC hg19) using a local installation of the Illumina DRAGEN pipeline. Data were analyzed using QIAGEN Clinical Insight Interpret in addition to an in-house variant interpretation pipeline. In addition, we used the DRAGEN copy number variant (CNV) pipeline to call CNV events using next-generation sequencing data; this involved a reference-based normalization algorithm that used additional matched normal samples to establish a baseline level from which CNV events were called. We also used the American College of Medical Genetics and Genomics (ACMG) guidelines for variant interpretation [15]. Positive CNV results (2 cases) were sent to a commercial laboratory for confirmation using a targeted single-nucleotide polymorphism array. The ClinVar and HGMD Professional 2023.2 databases were used to check for novelty of the detected variants.

### Sanger Sequencing

Sanger sequencing validation was performed to confirm the familial segregation of the detected variants in all family members. Oligonucleotide primers for polymerase chain reaction amplification of targeted variants were designed using Primer3Plus software (https://www.bioinformatics.nl/cgi-bin/primer3plus/primer3plus.cgi) and synthesized in-house. The amplified polymerase chain reaction products were sequenced using an ABI 3730xl capillary sequencer (Applied Biosystems, CA, USA), and the sequences were analyzed using Mutation Surveyor software V.3.24 (SoftGenetics LLC, State College, PA, USA).

## Results

**Figure 2.**
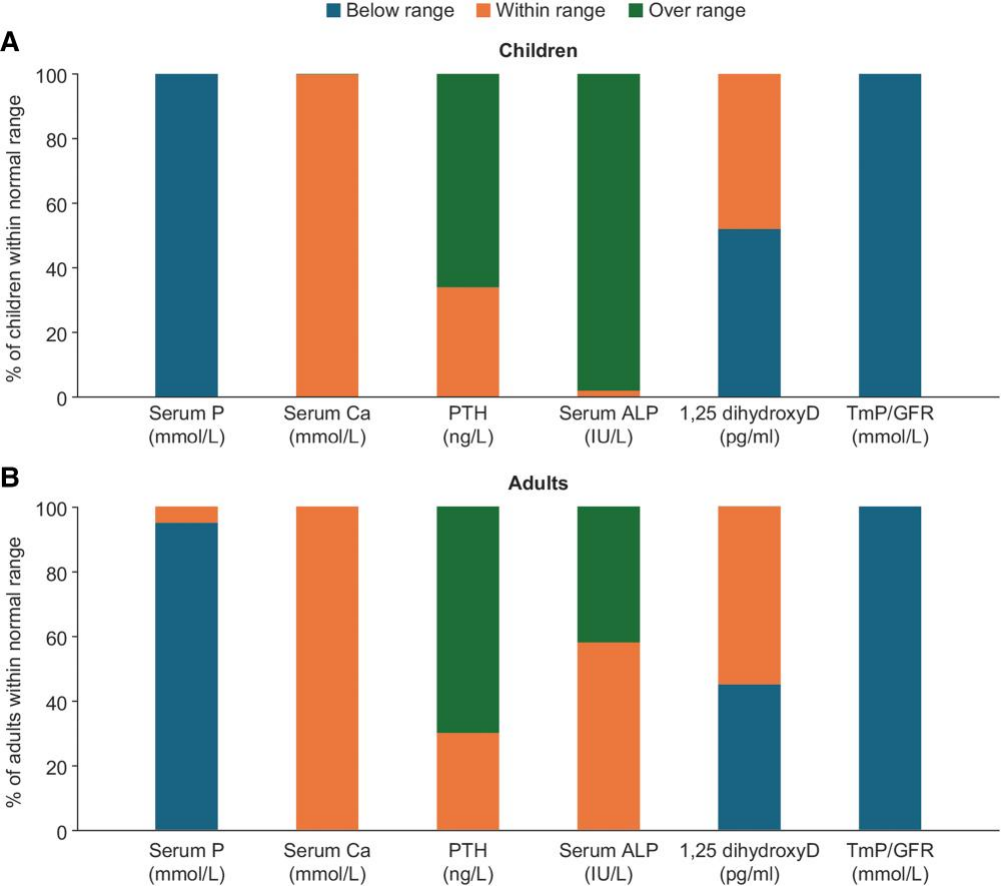
Summary of patients under/within/below normal ranges for biochemistry characteristics. (A) Children. (B) Adults. Abbreviations: ALP, alkaline phosphatase; Ca, calcium; GFR, glomerular filtration rate; P, phosphate; TmP, tubular maximum reabsorption of phosphate.

A total of 100 HR patients with symptoms/markers suggestive of XLH from 27 unrelated families were investigated. Consanguinity was reported in 20 families (74%) and absent in 7 families; a family history of HR was reported in 15 families. The cohort included 36 males and 64 females with an age range of 1.5 to 81 years and a median age of 23.5 years [interquartile range 9-42]. Among them, 43 patients were children (<18 years), and 57 were adults (≥18 years). Baseline characteristics are shown in Tables 1 and 2 and a summary of the percentage of patients below/within and above normal ranges for the biochemical characteristics is shown in Fig. 2.

**Table 2.** Summary of biochemistry characteristics at time of presentation

	Population	n	Reference range	Mean ± SD	Median ± SEM	Range
Serum P (mmol/L)	Children	43	Variable*^a^*	0.67 ± 0.11	0.65 ± 0.10	0.49-0.93
	Adults	56	Variable*^a^*	0.64 ± 0.08	0.64 ± 0.09	0.34-0.77
Serum Ca (mmol/L)	Children	43	2.1-2.6	2.31 ± 0.10	2.32 ± 0.35	2.12-2.63
	Adults	49	2.1-2.6	2.28 ± 0.10	2.29 ± 0.33	2.11-2.43
PTH (ng/L)	Children	41	15-65	60.90 ± 13.89	58.00 ± 9.51	32-92
	Adults	40	15-65	60.88 ± 13.12	59.50 ± 9.63	38-88
Serum ALP (IU/L)	Children	42	Variable*^a^*	579.26 ± 136.61	566.00 ± 89.38	146-876
	Adults	38	Variable*^a^*	129.26 ± 65.81	110.50 ± 20.97	70-345
1,25 dihydroxyD (pg/mL)	Children	42	48-192	47.10 ± 11.04	46.00 ± 7.27	23-94
	Adults	29	48-192	49.17 ± 13.91	48.00 ± 9.13	32-110
TmP/GFR (mmol/L)	Children	38	Variable*^a^*	0.66 ± 0.11	0.65 ± 0.11	0.45-0.89
	Adults	27	Variable*^a^*	0.63 ± 0.10	0.64 ± 0.12	0.45-0.84

Measurement	Age	Range	
		Male	Female
Serum P (mmol/L)	0-12	1.80-3.80	
	13 d-<1 y	1.36-2.26	
	1-<6 y	1.20-1.95	
	6-<11 y	1.00-1.75	
	11-<16 y	1.00-1.60	1.00-1.50
	16-<21 y	0.90-1.50	
	21-<31 y	0.80-1.45	0.90-1.50
	31 y+	0.70-1.45	0.80-1.45

Measurement	Age	Range	
		Male	Female
Serum ALP (IU/L)	0-14 d	83-248	
	15 d-1 y	122-469	
	1-<10 y	142-355	
	10-13	129-417	
	13-<15 y	116-468	57-254
	15-<17 y	82-331	50-117
	17-19 y	55-149	45-87
	>19 y	50-116	46-122

Measurement	Age	Range	
		Male	Female
TmP/GFR (mmol/L)	Birth-<3 mo	1.48-3.43	
	3-<6 mo	1.48-3.30	
	6 mo–<2 y	1.15-2.60	
	2–<15 y	1.15-2.24	
	15-<25 y	1.07-1.89	1.01-2.05
	25-45 y	1.00-1.35	0.96-1.44
	45-55 y	0.90-1.35	0.88-1.42
	65+ y	0.80-1.35	

Abbreviations: ALP, alkaline phosphatase; Ca, calcium; GFR, glomerular filtration rate; P, phosphate; SEM, standard error of the mean; TmP, tubular maximum reabsorption of phosphate.

^*a*^Reference range varies according to age and sex.

Clinical manifestations of HR were widespread, with 88% of children with short stature and 88% with bowing of the limbs. In adults, 98% were of short stature and 84% had bowing of the limbs. Craniosynostosis was noted in 30% of children compared with only 12% of adults. Overall, only 1 adult patient was asymptomatic. Disease severity varied, with only 2 patients (adults) classified as mild; more adults were classed as moderate than severe, but more children were classed as severe than moderate (Table 1). Phenotypic severity was variable even within the same family. Overall, of patients with disease rated as severe, 40% were male and 60% were female in children, and 53% and 47% were male and female, respectively, in adults. At the time of presentation, all children were treated with either conventional therapy (58%) or burosumab (42%). In contrast, 53% of adults were not receiving any treatment (this included the 4 deceased patients); of the 45% of adult patients receiving treatment, only 1 was receiving burosumab at the time of presentation.

WES detected clinically relevant variants in 23 families (Table 3). Pathogenic/likely pathogenic (P/LP) variants of HR were detected in 23 families with a diagnostic yield of 85% [20]. Most variants were detected in the *PHEX* gene (93 patients from 20 families); variants of the *ENPP1* gene were found in 2 families (2 patients); and 1 family (FAM-20) harbored a variant in the *DMP1* gene (1 patient); see Table 3. Overall, we detected 10 novel variants and predicted P/LP alleles. No genetic mutation was found in 4 families (FAM-15,18,19, and 21; Table 3). All 4 index patients had phenotypes suggestive of HR, 2 had a family history of consanguinity, and none had a family history of HR.

**Table 3.** HR families with detected gene variants

Family	Member	Gender	Age (y)	Serum P (mmol/L) (age- and sex-dependent range)	Serum Ca (mol/L) (normal range: 2.1-2.6)	PTH (ng/L) (normal range: 10-55 ng/L)	Serum ALP (IU/L) (age- and sex-dependent range)	1,25 dihydroxyD (pg/mlL (normal range: 48-192 pg/mL)	TmP/GFR (mmol/L) (age- and sex-dependent range)	Phenotype	Severity*^a^* (S, Mo, Mi)	Treatment (NT, C, B)	Alive/deceased	Gene
FAM-1 Consanguinity: Yes Family history: Yes	I:1	F	74	ND	ND	ND	ND	ND	ND	Short stature, bowed legs	Mo	NT	D	Gene: *PHEX* Genotype: NM_000444.6:c.2237G > T (p.Cys746Phe) Inheritance: XLD ACMG classification: PM1, PP2, PM2, PM5, PP3, PP5 (LP) Reference: rs1057517799
	II:1	M	78	0.55*^b^* (0.70-1.45)	2.34	56*^c^*	98 (50-116)	ND	ND	Short stature, bowed legs	S	NT	A	
	III:1	F	47	0.72*^b^* (0.80-1.45)	2.23	81*^c^*	70 (46-122)	51	ND	Asymptomatic	Mi	C	A	
	IV:4	M	16	0.83*^b^* (0.90-1.50)	2.38	55	495*^c^* (82-331)	56	0.75*^b^* (1.07-1.89)	Short stature, bowed legs, craniosynostosis	Mo	C	A	
	III:2	F	43	0.69*^b^* (0.80-1.45)	2.24	60*^c^*	113 (46-122)	42*^b^*	0.65*^b^* (0.96-1.44)	Short, obese, bowed legs, craniosynostosis	S	C	A	
	IV:8	M	13	0.68*^b^* (1.00-1.60)	2.34	92*^c^*	477*^c^* (116-468)	39*^b^*	0.74*^b^* (1.15-2.44)	Short stature, craniosynostosis	Mo	C	A	
	III:3	F	42	0.62*^b^* (0.70-1.45)	2.14	68*^c^*	88 (46-122)	43*^b^*	0.63*^b^* (0.96-1.44)	Short stature, bowed legs	Mo	C	A	
	III:4	F	39	0.60*^b^* (0.80-1.45)	2.12	56*^c^*	82 (46-122)	48	0.68*^b^* (0.96-1.44)	Short stature, bowed legs, craniosynostosis	S	C	A	
	IV:9	F	18	0.72*^b^* (0.90-1.50)	2.32	57*^c^*	136*^c^* (45-87)	52	0.55*^b^* (1.01-2.05)	Short stature, bowed legs	Mo	C	A	
	III:5	F	36	0.60*^b^* (0.80-1.45)	2.13	68*^c^*	93 (46-122)	54	0.67*^b^* (0.96-1.44)	Short stature, bowed legs, craniosynostosis	Mo	C	A	
	IV:11	M	9	0.70*^b^* (1.00-1.75)	2.35	54	367*^c^* (142-335)	57	0.76*^b^* (1.15-2.44)	Short stature, craniosynostosis	Mo	C	A	
	IV:12	M	6	0.93*^b^* (1.00-1.75)	2.39	75*^c^*	394*^c^* (142-335)	50	0.75*^b^* (1.15-2.44)	Craniosynostosis	Mo	C	A	
FAM-2 Consanguinity: Yes Family history: No	II:4	F	8	0.73*^b^* (1.00-1.75)	2.38	62*^c^*	616*^c^* (142-335)	43*^b^*	0.60*^b^* (1.15-2.44)	Short stature, bowing, craniosynostosis	S	B	A	Gene: *PHEX* Genotype: A deletion of exons 19 and 20 of PHEX Inheritance: XLD ACMG classification: LP Reference: Sarafrazi et al [16]
FAM-3 Consanguinity: No Family history: Yes	I:1	F	81	ND	ND	ND	ND	ND	ND	Short stature, low limb deformity	Mo	NT	D	Gene: *PHEX* Genotype: NM_000444.6: c.2148-10C > A Inheritance: XLD ACMG classification: PM2, PP3, PP1, PP4 (LP) [PMID: 38103548] Reference: Novel
	II:1	M	67	0.65*^b^* (0.70-1.45)	ND	ND	ND	ND	ND	Short stature, bowed legs	Mo	NT	A	
	II:2	M	66	0.72 (0.70-1.45)	ND	ND	ND	ND	ND	Short stature, bowed legs	Mo	NT	A	
	II:3	M	65	0.62*^b^* (0.70-1.45)	ND	ND	ND	ND	ND	Short stature, bowed legs	Mo	NT	A	
	III:1	F	39	0.73*^b^* (0.80-1.45)	ND	ND	ND	ND	ND	Short stature, bowed legs	S	C	A	
	III:2	F	38	0.59*^b^* (0.80-1.45)	ND	ND	ND	ND	ND	Short stature, bowed legs	Mo	C	A	
	III:3	F	36	0.77*^b^* (0.80-1.45)	ND	ND	ND	ND	ND	Short stature, bowed legs	Mo	C	A	
	IV:1	F	3	0.65*^b^* (1.20-1.95)	2.38	68*^c^*	554*^c^* (142-335)	44*^b^*	0.53*^b^* (1.15-2.44)	Short stature, bilateral bowing deformity, craniosynostosis	S	B	A	
	IV:2	F	3	0.53*^b^* (1.2-1.95)	2.43	72*^c^*	567*^c^* (142-335)	38*^b^*	0.45*^b^* (1.15-2.44)	Short stature, bilateral bowing deformity, craniosynostosis	S	B	A	
	IV:3	M	7	0.49*^b^* (1.00-1.75)	2.28	55	678*^c^* (142-335)	51	0.51*^b^* (1.15-2.44)	Short stature, bowed legs, craniosynostosis	S	B	A	
FAM-4 Consanguinity: Yes Family history: Yes	I:1	M	78	0.65*^b^* (0.70-1.45)	2.25	ND	ND	ND	ND	Short stature, lower limbs bowing, wheelchair-bound	Mo	NT	A	Gene: *PHEX* Genotype: NM_000444.6:c.1699C > T (p.Arg567Ter) Inheritance: XLD ACMG classification: PVS1, PM2, PP5 (P) Reference: rs137853271
	II:2	F	45	0.66*^b^* (0.80-1.45)	2.34	ND	ND	ND	ND	Short stature, lower limb deformities	Mo	NT	A	
	II:1	F	42	0.59*^b^* (0.80-1.45)	2.29	ND	ND	ND	ND	Short stature, craniosynostosis	Mo	NT	A	
	III:1	M	3	0.65*^b^* (1.2-1.95)	2.32	56*^c^*	498*^c^* (142-335)	39*^b^*	0.54*^b^* (1.15-2.44)	Short stature, craniosynostosis	S	B	A	
FAM-5 Consanguinity: Yes Family history: Yes	I:1	F	26	0.65*^b^* (0.90-1.50)	2.25	ND	ND	110*^c^*	ND	Short stature, bowed legs	Mo	NT	A	Gene: *PHEX* Genotype: NM_000444.6:c.1645C > T (p.Arg549Ter) Inheritance: XLD ACMG classification: PVS1, PM2, PP5 (P) Reference: rs886041224
	II:1	M	5	0.59*^b^* (1.20-1.95)	2.38	67*^c^*	567*^c^* (142-335)	54	0.71*^b^* (1.15-2.44)	Short stature, bowed legs, craniosynostosis	S	B	A	
	II:2	F	3	0.67*^b^* (1.2-1.95)	2.29	54	488*^c^* (142-335)	49	0.56*^b^* (1.15-2.44)	Short stature, bowed legs, craniosynostosis	S	B	A	
FAM-6 Consanguinity: No Family history: Yes	II:1	F	74	0.55*^b^* (0.80-1.45)	2.21	73*^c^*	88 (46-122)	ND	ND	Short stature, bowing of legs with multiple surgeries, limited movement, walking aids	S	NT	D	Gene: *PHEX* Genotype: NM_000444.6:c.1645C > T (p.Arg549Ter) Inheritance: XLD ACMG classification: PVS1, PM2, PS2 (P) Reference: rs886041227
	II:2	F	73	0.67*^b^* (0.80-1.45)	2.34	62*^c^*	93 (46-122)	ND	ND	Short with bowing of legs with multiple surgeries, limitation of movement with using walking aids	S	NT	D	
	III:1	M	38	0.63*^b^* (0.70-1.45)	2.25	45	86 (50-116)	ND	ND	Short stature, bowing of legs with multiple surgeries, limited movement, walking aids	S	C	A	
	III:2	M	41	0.56*^b^* (0.70-1.45)	2.18	56*^c^*	86 (50-116)	ND	ND	Short stature, bowing of legs with multiple surgeries, limited movement, walking aids	S	C	A	
	III:4	M	45	0.74 (0.70-1.45)	2.34	78*^c^*	100 (50-116)	ND	ND	Short stature, bowing of legs with multiple surgeries, limited movement, walking aids	S	C	A	
	III:5	F	55	0.75*^b^* (0.80-1.45)	2.36	69*^c^*	93 (46-122)	48	0.65*^b^* (0.88-142)	Short stature, bowing of legs with multiple surgeries, limited movement, walking aids	S	C	A	
	III:6	M	48	0.61*^b^* (0.70-1.45)	2.33	88*^c^*	136*^c^* (50-116)	52	0.45*^b^* (0.90-1.35)	Short stature, bowing of legs with multiple surgeries, limited movement, walking aids	S	C	A	
	III:7	M	47	0.74 (0.70-1.45)	2.38	63*^c^*	118*^c^* (50-116)	35*^b^*	0.55*^b^* (0.90-1.35)	Short stature, bowing of legs with multiple surgeries, limited movement, walking aids	Mo	NT	A	
	III:8	F	43	0.59*^b^* (0.80-1.45)	2.16	43	88 (46-122)	47*^b^*	0.62*^b^* (0.96-1.44)	Short stature, bowed legs	Mo	NT	A	
	III:9	F	38	0.71*^b^* (0.80-1.45)	2.25	54	96 (46-122)	55	0.53*^b^* (0.9-1.44)	Short stature, bowing of legs with multiple surgeries	Mo	NT	A	
	III:10	M	35	0.69*^b^* (80.8-1.45)	2.37	67*^c^*	108 (50-116)	54	0.65*^b^* (1.00-1.35)	Short stature, bowing of legs with multiple surgeries	Mo	NT	A	
	III:11	M	41	0.64*^b^* (0.80-1.45)	2.41	49	84 (50-116)	41*^b^*	0.46*^b^* (1.00-1.35)	Short stature, bowing of legs with multiple surgeries	Mo	NT	A	
	III:12	M	43	0.56*^b^* (0.80-1.45)	2.19	56*^c^*	79 (50-116)	32*^b^*	0.64*^b^* (1.00-1.35)	Short stature, bowed legs	Mo	NT	A	
	III:13	M	29	0.59*^b^* (0.80-1.45)	2.34	67*^c^*	117*^c^* (50-116)	45*^b^*	0.63*^b^* (1.00-1.35)	Short stature, bowing of legs with multiple surgeries, limited movement, walking aids	Mo	NT	A	
	III:14	F	33	0.56*^b^* (0.80-1.45)	2.23	54	121 (46-122)	46*^b^*	0.66*^b^* (0.96-1.44)	Short stature, bowed legs	Mo	NT	A	
	IV:1	F	11	0.64*^b^* (1.00-1.50)	2.36	49	497*^c^* (129-417)	53	0.72*^b^* (1.15-2.44)	Short stature, bowed legs, craniosynostosis	Mo	C	A	
	IV:2	F	7	0.63*^b^* (1.00-1.75)	2.21	56*^c^*	545*^c^* (142-335)	38*^b^*	0.61*^b^* (1.15-2.44)	Short stature, bowed legs, craniosynostosis	Mo	C	A	
	IV:3	F	6	0.78*^b^* (1.00-1.75)	2.17	69*^c^*	475*^c^* (142-335)	45*^b^*	0.59*^b^* (1.15-2.44)	Short stature, bowed legs	Mo	C	A	
	IV:6	M	25	0.76*^b^* (0.80-1.45)	2.37	73*^c^*	119*^c^* (50-116)	34*^b^*	0.78*^b^* (1.00-1.35)	Short stature, bowed legs	Mo	C	A	
	IV:7	M	34	0.59*^b^* (0.70-1.45)	2.25	65*^c^*	233*^c^* (50-116)	53	0.52*^b^* (1.00-1.35)	Short stature, bowed legs	Mo	C	A	
	IV:8	F	21	0.67*^b^* (0.90-1.50)	2.13	69*^c^*	133*^c^* (46-122)	45*^b^*	0.73*^b^* (1.01-2.05)	Short stature, bowed legs	Mo	C	A	
	IV:11	F	20	0.64*^b^* (0.90-1.50)	2.15	49	156*^c^* (46-122)	59	0.64*^b^* (1.01-2.05)	Short stature, bowed legs	Mo	C	A	
	IV:14	F	25	0.72*^b^* (0.90-1.50)	2.43	74*^c^*	176*^c^* (46-122)	54	0.83*^b^* (0.96-1.44)	Short stature, bowed legs	Mo	C	A	
	IV:15	F	22	0.62*^b^* (0.90-1.50)	2.37	59*^c^*	168*^c^* (46-122)	59	0.69*^b^* (0.96-1.44	Short stature, bowed legs	Mo	C	A	
	IV:16	F	16	0.66*^b^* (0.90-1.50)	2.24	62*^c^*	146*^c^* (50-117)	56	0.74*^b^* (1.01-2.05)	Short stature, bowed legs	Mo	C	A	
	IV:17	F	11	0.78*^b^* (1.00-1.50)	2.17	57*^c^*	367 (129-417)	35*^b^*	0.65*^b^* (1.15-2.44)	Short stature, bowed legs	Mo	C	A	
FAM-7 Consanguinity: No Family history: Yes	II:1	M	39	0.62*^b^* (0.70-1.45)	2.38	74*^c^*	98 (50-116)	47*^b^*	0.54*^b^* (1.00-1.35)	Short stature, bowed legs	S	B	A	Gene: *PHEX* Genotype: NM_000444.6:c.292dup (p.Met98AsnfsTer13) Inheritance: XLD ACMG classification: PVS1, PM2 (LP) Reference: Novel
	II:2	F	11	0.54*^b^* (1.00-1.50)	2.21	66*^c^*	532*^c^* (129-417)	45*^b^*	0.67*^b^* (1.15-2.44)	Short stature, bowed legs	S	B	A	
	II:3	F	2	0.71*^b^* (1.20-1.95)	2.26	43	658*^c^* (142-335)	51	0.89*^b^* (1.15-2.44)	Short stature, bowed legs	S	B	A	
FAM-8 Consanguinity: No Family history: Yes	II:1	F	45	0.54*^b^* (0.80-1.45)	2.43	45	87 (46-122)	35*^b^*	0.65*^b^* (0.80-1.42)	Short stature, bowed legs	Mo	NT	A	Gene: *PHEX* Genotype: NM_000444.6:c.1241del (p.Leu414ProfsTer10) Inheritance: XLD ACMG classification: PVS1, PM2, PP5 (P) Reference: rs886041446
	III:4	M	18	0.69*^b^* (0.90-1.50)	2.38	56*^c^*	299*^c^* (55-149)	43*^b^*	0.84*^b^* (1.07-1.89)	Short stature, bowed legs	S	C	A	
	III:5	M	12	0.59*^b^* (1.00-1.60)	2.12	72*^c^*	544*^c^* (129-417)	56	0.78*^b^* (1.15-2.24)	Short stature, bowed legs	S	C	A	
	III:6	M	6	0.62*^b^* (1.00-1.75)	2.26	63*^c^*	743*^c^* (142-335)	36*^b^*	0.62*^b^* (1.15-2.44)	Short stature, bowed legs	Mo	C	A	
FAM-9 Consanguinity: No Family history: Yes	II:1	F	38	0.52*^b^* (0.80-1.45)	2.28	57*^c^*	132*^c^* (46-122)	52	0.63*^b^* (0.96-1.44)	Short stature, bowed legs	S	C	A	Gene: *PHEX* Genotype: NM_000444.6:c.846del (p.Glu283ArgfsTer2) Inheritance: XLD ACMG classification: PVS1, PM2 (LP) Reference: Novel
	III:3	M	8	0.55*^b^* (1.00-1.75)	2.14	43	517*^c^* (142-335)	35*^b^*	0.76*^b^* (1.15-2.44)	Short stature, genu valgus, craniosynostosis	S	C	A	
	III:4	F	10	0.75*^b^* (1.00-1.75)	2.35	75*^c^*	788*^c^* (129-147)	50	0.87*^b^* (1.15-2.44)	Short stature, genu valgus, craniosynostosis	S	C	A	
FAM-10 Consanguinity: Yes Family history: Yes	I:1	F	42	0.57*^b^* (0.80-1.45)	2.23	85*^c^*	132*^c^* (46-122)	ND	ND	Short stature, bowed legs	Mo	NT	A	Gene: *PHEX* Genotype: NM_000444.6:c.1318G > T (p.Glu440Ter) Inheritance: XLD ACMG classification: PVS1, PM2 (LP) Reference: Novel
	II:1	F	11	0.59*^b^* (1.00-1.50)	2.31	54	597*^c^* (129-417)	42*^b^*	0.63*^b^* (1.15-2.44)	Short stature, bowed legs	S	C	A	
	II:2	M	22	0.34*^b^* (0.80-1.45)	2.18	41	345*^c^* (50-116)	36*^b^*	0.59*^b^* (1.00-1.35	Short stature, genu valgus, craniosynostosis	S	C	A	
FAM-11 Consanguinity: Yes Family history: Yes	II:1	M	45	0.59*^b^* (0.70-1.45)	2.34	63*^c^*	89 (50-116)	ND	ND	Short stature, bowed legs	Mo	NT	A	Gene: *PHEX* Genotype: A duplication including exon 17 of PHEX Inheritance: XLD ACMG classification: LP Reference: Novel
	III:1	F	13	0.71*^b^* (1.00-1.50)	2.26	84*^c^*	674*^c^* (57-254)	45*^b^*	0.52*^b^* (1.15-2.44)	Short stature, bowed legs	Mo	C	A	
FAM-12 Consanguinity: Yes Family history: Yes	II:1	F	35	0.68*^b^* (0.80-1.45)	2.35	42	121 (46-122)	ND	ND	Short stature	Mi	NT	A	Gene: *PHEX* Genotype: NM_000444.6:c.1966-1G > T (splicing defect) Inheritance: XLD ACMG classification: PVS1, PM2, PP5 (LP) Reference: Marik et al [17]
	III:2	M	2	0.64*^b^* (1.20-1.95)	2.16	54	745*^c^* (142-335)	50	0.56*^b^* (1.15-2.44)	Short stature, bowed legs	S	B	A	
FAM-13 Consanguinity: Yes Family history: No	II:1	F	5	0.53*^b^* (1.20-1.95)	2.23	43	612*^c^* (142-335)	48	0.51*^b^* (1.15-2.44)	Short stature, bowed legs	S	C	A	Gene: *PHEX* Genotype: NM_000444.6:c.188-2A > C (Splicing defect) Inheritance: XLD ACMG classification: PVS1, PM2 (LP) Reference: Rush et al [3]
FAM-14 Consanguinity: Yes Family history: Yes	II:1	F	48	0.59*^b^* (0.80-1.45)	2.29	ND	ND	ND	ND	Short stature, bowed legs	Mo	NT		Gene: *PHEX* Genotype: NM_000444.6:c.1701A > C (p.Arg567=) Inheritance: XLD ACMG classification: PM2, PP5 (LP) Reference: Novel
	III:1	M	16	0.69*^b^* (0.90-1.50)	2.32	ND	ND	ND	ND	Short stature, bowed legs	S	C	A	
	III:2	F	9	0.62*^b^* (1.00-1.75)	2.38	69*^c^*	668*^c^* (142-355)	51	ND	Short stature, bowed legs	S	C	A	
	III:3	F	2	0.81*^b^* (1.20-1.95)	2.21	78*^c^*	565*^c^* (142-335)	42*^b^*	ND	Short stature, genu valgus, craniosynostosis	S	C	A	
FAM-15 Consanguinity: Yes Family history: No	II:1	M	9	0.62*^b^* (1.00-1.75)	2.41	62*^c^*	479*^c^* (142-335)	53	0.72*^b^* (1.15-2.44)	Short stature, bowed legs	S	B	A	Negative
FAM-16 Consanguinity: Yes Family history: No	II:1	F	8	0.53*^b^* (1.00-1.75)	2.12	54	734*^c^* (142-335)	47*^b^*	0.58*^b^* (1.15-2.44)	Short stature, bowed legs	Mo	C	A	Gene: *PHEX* Genotype: NM_000444.6:c.1536T > G (p.Tyr512Ter) Inheritance: XLD ACMG classification: PVS1, PM2 (LP) Reference: Novel
FAM-17 Consanguinity: Yes Family history: No	II:1	F	12	0.59*^b^* (1.00-1.50)	2.36	58*^c^*	762*^c^* (129-417)	32*^b^*	0.71*^b^* (1.15-2.44)	Short stature, bowed legs	S	C	A	Gene: *PHEX* Genotype: NM_000444.6: c.871C > T (p.Arg291Ter) Inheritance: XLD ACMG classification: PVS1, PS4, PM2 (P) Reference: rs866429868
FAM-18 Consanguinity: Yes Family history: No	II:1	F	14	0.59*^b^* (1.00-1.50)	2.25	71*^c^*	584*^c^* (57-254)	39*^b^*	0.48*^b^* (1.15-2.44)	Short stature, bowed legs	S	C	A	Negative
FAM-19 Consanguinity: No Family history: No	I:1	F	14	0.61*^b^* (1.00-1.50)	2.29	89*^c^*	659*^c^* (57-254)	45*^b^*	0.62*^b^* (1.15-2.44)	Short stature, bowed legs	S	C	A	Negative
FAM-20 Consanguinity: No Family history: No	I:1	M	4	0.53*^b^* (1.20-1.95)	2.21	59*^c^*	572*^c^* (142-335)	68	0.68*^b^* (1.15-2.44)	Short stature, bowed legs	S	C	A	Gene: *DMP1* Genotype: PVS1, PS1, PM2, PP5 (P) Inheritance: AR ACMG classification: PVS1, PS4, PM2 (P) Reference: rs104893834
FAM-21 Consanguinity: Yes Family history: No	I:1	M	20	0.59*^b^* (0.90-1.50)	2.14	38	324*^c^* (50-116)	54	0.54*^b^* (1.07-1.89)	Short stature with bowed legs	S	C	A	Negative
FAM-22 Consanguinity: Yes Family history: No	I:1	F	5	0.67*^b^* (1.20-1.95)	2.63*^c^*	56*^c^*	792*^c^* (142-335)	94	0.58*^b^* (1.15-2.44)	Short stature, genu valgus	S	C	A	Gene: *PHEX* Genotype: NM_000444.6:c.208_212del (p.Val70SerfsTer7) Inheritance: XLD ACMG classification: PVS1, PS2, PM2 (P) Reference: Hernández-Frías et al [18]
FAM-23 Consanguinity: Yes Family history: Yes	I:1	F	72	0.57*^b^* (0.80-1.45)	2.13	ND	ND	ND	ND	Short stature, bowed legs	Mo	NT	A	Gene: *PHEX* Genotype: NM_000444.6: c.2104C > T (p.Arg702Ter) Inheritance: XLD ACMG classification: PS4, PVS1, PM2 (LP) Reference: Lin et al [19]
	II:1	F	42	0.68*^b^* (0.80-1.45)	2.39	ND	ND	ND	ND	Short stature, bowed legs	Mo	NT	A	
	II:2	F	40	0.71*^b^* (0.80-1.45)	2.21	ND	ND	ND	ND	Short stature, bowed legs	Mo	NT	A	
	II:3	M	39	0.59*^b^* (0.70-1.45)	2.31	ND	ND	ND	ND	Short stature, bowed legs	Mo	NT	A	
	III:1	F	10	0.68*^b^* (1.00-1.75)	2.38	47	463*^c^* (129-417)	53	0.68*^b^* (1.15-2.44)	Short stature, bowed legs	Mo	B	A	
	III:2	F	3	0.91*^b^* (1.20-1.95)	2.45	32	545*^c^* (142-335)	49	0.84*^b^* (1.15-2.44)	Short stature, bowed legs	Mo	B	A	
FAM-24 Consanguinity: Yes Family history: Yes	I:1	F	68	0.67*^b^* (0.8-1.45)	2.43	39	ND	ND	ND	Short stature, bowed legs	Mo	NT	A	Gene: *PHEX* Genotype: NM_000444.6: c.436 + 4A > G Inheritance: XLD ACMG classification: PM2, PP3, PP5 (LP) Reference: rs1057520344
	II:1	F	37	0.56*^b^* (0.80-1.45)	2.35	52	ND	ND	ND	Short stature, bowed legs	Mo	C	A	
	III:1	F	10	0.71*^b^* (1.00-1.75)	2.29	44	545*^c^* (129-417)	45*^b^*	0.58*^b^* (1.15-2.44)	Short stature, bowed legs	Mo	B	A	
	III:2	F	1.5	0.89*^b^* (1.20-1.95)	2.45	86*^c^*	876*^c^* (142-355)	51	0.79*^b^* (1.15-2.44)	Bowed legs	Mo	B	A	
FAM-25 Consanguinity: Yes Family history: Yes	I:1	F	35	0.63*^b^* (0.80-1.45)	2.11	84*^c^*	127*^c^* (46-122)	ND	ND	Short stature, bowed legs	Mo	C	A	Gene: *PHEX* Genotype: NM_000444.6:c.1174-10_1174-1del Inheritance: XLD ACMG classification: PVS1, PM2 (LP) Reference: Novel
	II:1	F	1.5	0.79*^b^* (1.20-1.95)	2.34	56*^c^*	756*^c^* (142-355)	42*^b^*	0.65*^b^* (1.15-2.44)	Bowed legs	Mo	B	A	
FAM-26 Consanguinity: Yes Family history: No	II:1	**F**	9	0.65*^b^* (1.00-1.75)	2.37	35	543*^c^* (142-335)	23*^b^*	ND	Bowed legs	Mo	B	A	Gene: *ENPP1* Genotype: NM_000444.6:c.1174-10_1174-1del NM_006208.3:c.195_197delinsAA (p.Ala66ThrfsTer22) Inheritance: AR ACMG classification: PVS1, PM2 (LP) PVS1, PM2 (LP) Reference: Novel
FAM-27 Consanguinity: Yes Family history: No	II-1	F	14	0.71*^b^* (1.00-1.50)	2.25	57*^c^*	645*^c^* (57-254)	39*^b^*	ND	Bowed legs	Mo	B	A	Gene: *ENPP1* Genotype: NM_006208.3:c.1724-1G > T Inheritance: AR ACMG classification: PVS1, PM2 (LP) Reference: Novel

Abbreviations: 1,25 dihydroxyD, 1,25 dihydroxyvitamin D; A, alive; ACMG, American College of Medical Genetics and Genomics; ALP, alkaline phosphatase; AR, autosomal recessive; B, burosumab, C, conventional; D, deceased, F, female; GFR, glomerular filtration rate; LP, likely pathogenic; M, male; Mi, mild; Mo, moderate; ND, not done; NT, not treated; P, pathogenic; S, severe; serum P, serum phosphate; TmP, tubular maximum reabsorption of phosphate; VUS, clinically relevant variant of uncertain significance; XLD, X-linked dominant.

^*a*^Disease severity was defined as: mild: only biochemical abnormalities suggestive of hypophosphatemic rickets (ie, low phosphate and high alkaline phosphatase for age and gender) and otherwise asymptomatic with no skeletal deformity. Moderate: biochemical abnormalities suggestive of hypophosphatemic rickets with skeletal deformity. Severe: biochemical abnormalities suggestive of hypophosphatemic rickets with skeletal deformity and pain with limitation in mobility ± using a mobility assistance device.

^*b*^Below normal range.

^*c*^Over normal range.

The majority of the *PHEX*-detected variants in our study were loss of function variants identified in 19 of the molecular genetically solved families with P/LP variants, whereas previously reported missense variants were detected in Family-11 (NM_000444.6:c.2237G > T; p.Cys746Phe). The *PHEX* variant: c.1701A > C (p.Arg567=) in Family-14 is considered to be exonic splicing and is expected to lead to loss of protein function. The variant *PHEX*: c.2148-10C > A in Family-3 (FAM-3) is considered to be a splicing variant and is expected to lead to loss of function. Both variants detected in the *ENPP1* gene c.195_197delinsAA (FAM-26) and c.1724-1G > T (FAM-27) are expected to cause ENPP1 deficiency. The causative variant of HR in Family-20 was detected in the *DMP1* gene. The variant NM_004407.4: c.1A > G (p.Met1?) has a start-loss effect, leading to the reduction or elimination of protein production. X-linked dominant inheritance was the major mode of inheritance in our cohort because of *PHEX* variants located on the X-chromosome.

## Discussion

XLH is a rare inherited metabolic bone disease with an estimated incidence of 1 per 20 000 live births worldwide [21]. The prevalence of rickets in Saudi Arabia was estimated at 15.3% in 2018 [22]. A local study based on clinical data [2] reported inherited rickets and osteomalacia to account for approximately 4% of all reported cases of rickets in a Saudi population. However, estimating the prevalence based on clinical data could underestimate the actual patient population in Saudi Arabia, and it is more appropriate to estimate prevalence based on genetic findings [23]. Our study is a step toward providing accurate prevalence data for XLH in our population.

Our HR cohort of 27 families revealed a diverse clinical and demographic profile, and we detected a diagnostic (P/LP) molecular genetic variant in 85% of families. *PHEX* is expressed in osteocytes and odontoblasts [24], and deficiency of *PHEX* expression leads to elevated *FGF23* expression and production of intact FGF23 [25]. Most of the *PHEX* variants detected in our cohort were loss of function variants that may lead to functional variation in *PHEX* protein activity.

Although the number of genes that cause HR are limited, they all represent Mendelian inheritance. Defects in *DMP1*, *FAM20C*, and *ENPP1* genes cause autosomal recessive HR, whereas *FGF23* variants cause autosomal dominant HR. The most common cause of inherited rickets is caused by X-linked dominant variants in the *PHEX* gene. In our cohort, most variants detected were in the *PHEX* gene except for 3 families who harbored variants in *DMP1* and *ENPP1*. In the 4 families (FAM-15,18,19, and 21) in which no genetic mutation was found despite highly suggestive phenotypes, it is possible that these were novel mutations that were not detected by currently available microarrays [3]. Although the next-generation sequencing gene panel would be sufficient for genetic diagnosis of HR, we believe that WES and whole-genome sequencing may suggest a diagnostic method not previously considered.

Although the clinical manifestations of XLH persist throughout a lifetime, the standard clinical practice in many countries is to discontinue treatment when skeletal growth is complete and only resume when symptoms present in adulthood, which often results in a gap in care [12]. Hypophosphatemia and elevated ALP, biochemical alterations that should prompt a diagnostic workup in adult patients [26] were apparent in 100% and approximately one-third of our adult patients, respectively. In many Gulf countries, the transition to adult services begins at approximately 14 years of age [8]. The efficacy of conventional therapy in adults with XLH is unclear but there is a growing body of evidence to support the use of burosumab in adults to restore phosphate homeostasis, improve quality of life and improve the healing of fractures [27-31]. Recommendations for the optimal treatment of people with XLH from adolescence to adulthood are likely to be updated as additional clinical data and real-world experience with burosumab are published, and a more integrated, life course approach [6] can be developed.

Burosumab treatment has demonstrated clinical improvement of rickets and growth in children with XLH in comparison with conventional therapy [25]. It is known that early diagnosis and treatment are associated with better outcomes in children [6]. Because XLH children are not born with rickets but develop signs of it over several months [32], genetic testing can detect molecular alterations in the prenatal and neonatal period of life and, in families with history of XLH, enable early treatment with burosumab to optimize outcomes. By identifying types of HR independent of FGF23, genetic testing can also enable early treatment with the recommended therapy [33]. A study in 2 adult patients with autosomal recessive HR resulting from mutations in the *DMP1* gene suggested potential benefits of adding burosumab to the treatment protocol [34]. For HR associated with *ENPP1* variants, where burosumab is not considered an option [35], treatments with oral phosphate and calcitriol are recommended [36]. Currently, ENPP1 replacement therapy is being investigated in animal models [37, 38].

**Figure 3.**
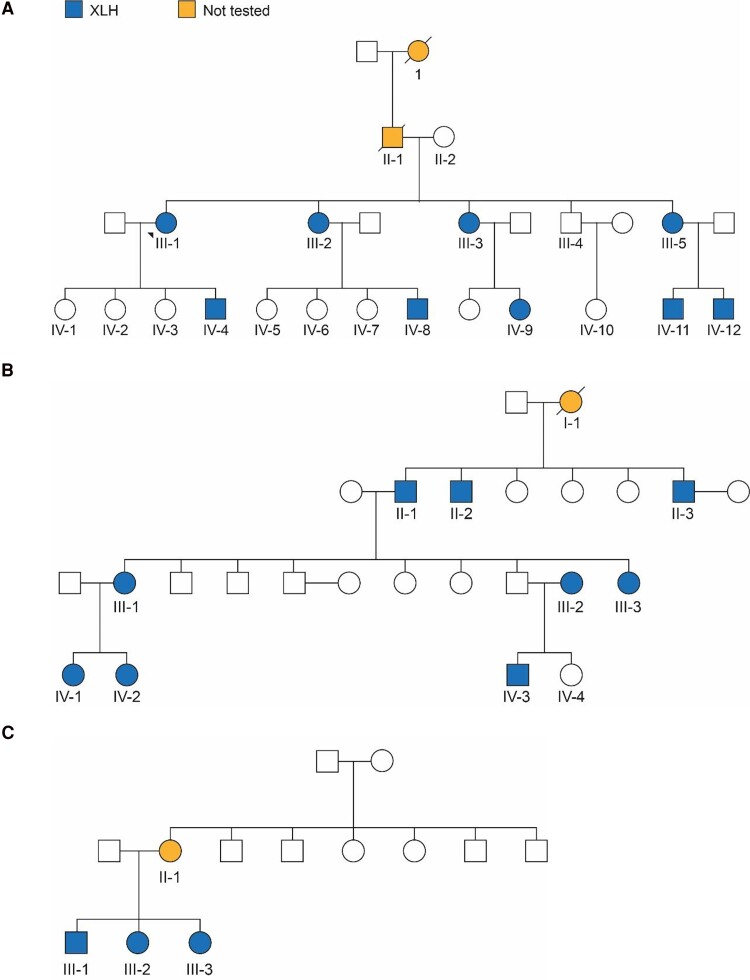
Pedigree analysis for three families with XLH. (A) Family 1: heterozygous for c.2237 G > T (p.C746F). (B) Family 3: heterozygous for c.2148-10 C > A. (C) Family 7: heterozygous PHEX c.292dup (p.Met98AsnfsTer13). Abbreviations: PHEX, phosphate regulating endopeptidase X-linked; XLH, X-linked hypophosphatemia.

Because *PHEX* is the only known gene to cause XLH, pedigree analysis can help healthcare providers to anticipate the need for counseling and treatment related to XLH, especially in large families such as FAM-1, FAM-3, and FAM-7 (see Fig. 3). The pedigree analysis and genetics results in this study will be used to enhance counseling to prevent transmission of the disease in future generations, including offering prenatal counseling, preimplantation genetic testing, and informed choices on management of affected pregnancies.

Establishing phenotype–genotype correlation in XLH is still challenging, and the association between the severity of XLH phenotype and the type of mutation is controversial [39]. A trend has suggested greater skeletal severity in *PHEX* truncation variant and the location of the variant [40, 41], whereas others have suggested no correlation [42]. In our cohort, we were unable to establish a solid relationship between severity of the disease and type of variants detected. In fact, we observed variation in severity within the same family. In large families such as FAM-3 and FAM-6, the severity of disease ranged between moderate and severe within the family and can be attributed to the individual's genetic background. This study is limited to patients already under care at 1 center in Saudi Arabia, and their families and cannot therefore provide broader population information about the prevalence or characteristics of XLH.

In conclusion, this was the first study in the region to identify the molecular genetics and incidence of XLH in patients from a large hospital in Saudi Arabia. The study provided clinical and molecular insights that increase our understanding of the pathogenesis of XLH and highlighted the underdiagnosis of adult XLH in our population. Because most family members were diagnosed after the pedigree analysis, we suggest that pedigree analysis could be used in combination with genetic findings to provide genetic counseling and facilitate early diagnosis to improve long-term outcomes.

## Data Availability

Original data generated and analyzed during this study are included in this published article. The detected variants were submitted to ClinVar (clinvar@ncbi.nlm.nih.gov) with submission ID: SUB14151291.

## Abbreviations

ACMG: American College of Medical Genetics and Genomics
ALP: alkaline phosphatase
CNV: copy number variant
FGF23: fibroblast growth factor 23
HR: hypophosphatemic rickets
KFSHRC: King Faisal Specialist Hospital and Research Centre
P/LP: pathogenic/likely pathogenic
WES: whole-exome sequencing
XLH: X-linked hypophosphatemia
